# Chronic obstructive pulmonary disease prevalence and prediction in a high-risk lung cancer screening population

**DOI:** 10.1186/s12890-020-01344-y

**Published:** 2020-11-16

**Authors:** John R. Goffin, Gregory R. Pond, Serge Puksa, Alain Tremblay, Michael Johnston, Glen Goss, Garth Nicholas, Simon Martel, Rick Bhatia, Geoffrey Liu, Heidi Schmidt, Sukhinder Atkar-Khattra, Annette McWilliams, Ming-Sound Tsao, Martin C. Tammemagi, Stephen Lam

**Affiliations:** 1 Department of Oncology, McMaster University, Juravinski Cancer Centre, 699 Concession St., Hamilton, ON L8V 5C2 Canada; 2 University of Calgary, 3300 Hospital Drive NW, Calgary, AB T2N 4N1 Canada; 3 Dalhousie University, 5850 College St, PO Box 15000, Halifax, NS B3J 3Z3 Canada; 4 Ottawa Hospital Research Institute, University of Ottawa, 501 Smyth Rd, Box 511, Ottawa, ON K1H 8L6 Canada; 5 Centre de recherche de l’Institut universitaire de cardiologie et pneumonolgie de Québec, Université Laval, QC, Québec G1V 4G5 Canada; 6 Health Sciences Centre - General Hospital, Memorial University, 300 Prince Phillip Dr, St. John’s, NF A1B 3V6 Canada; 7 University Health Network and Princess Margaret Cancer Centre, 610 University Ave, Toronto, ON M5G 2M9 Canada; 8 British Columbia Cancer Research Centre, University of British Columbia, 675 West 10th Ave, Vancouver, BC V5Z 1L3 Canada; 9 Fiona Stanley Hospital, University of Western Australia, 11 Robin Warren Dr, Murdoch, W Australia 6150 Australia; 10 Department of Health Sciences, Brock University, Walker Complex South, Rm 306, 500 Glenridge Ave, St. Catharines, ON L2S 3A1 Canada

**Keywords:** Lung cancer, Screening, Chronic obstructive pulmonary disease, Spirometry, CT scan

## Abstract

**Background:**

Chronic obstructive pulmonary disease (COPD) is an underdiagnosed condition sharing risk factors with lung cancer. Lung cancer screening may provide an opportunity to improve COPD diagnosis. Using Pan-Canadian Early Detection of Lung Cancer (PanCan) study data, the present study sought to determine the following: 1) What is the prevalence of COPD in a lung cancer screening population? 2) Can a model based on clinical and screening low-dose CT scan data predict the likelihood of COPD?

**Methods:**

The single arm PanCan study recruited current or former smokers age 50–75 who had a calculated risk of lung cancer of at least 2% over 6 years. A baseline health questionnaire, spirometry, and low-dose CT scan were performed. CT scans were assessed by a radiologist for extent and distribution of emphysema. With spirometry as the gold standard, logistic regression was used to assess factors associated with COPD.

**Results:**

Among 2514 recruited subjects, 1136 (45.2%) met spirometry criteria for COPD, including 833 of 1987 (41.9%) of those with no prior diagnosis, 53.8% of whom had moderate or worse disease. In a multivariate model, age, current smoking status, number of pack-years, presence of dyspnea, wheeze, participation in a high-risk occupation, and emphysema extent on LDCT were all statistically associated with COPD, while the overall model had poor discrimination (c-statistic = 0.627 (95% CI of 0.607 to 0.650). The lowest and the highest risk decile in the model predicted COPD risk of 27.4 and 65.3%.

**Conclusions:**

COPD had a high prevalence in a lung cancer screening population. While a risk model had poor discrimination, all deciles of risk had a high prevalence of COPD, and spirometry could be considered as an additional test in lung cancer screening programs.

**Trial registration:**

(Clinical Trial Registration: ClinicalTrials.gov, number NCT00751660, registered September 12, 2008)

**Supplementary Information:**

The online version contains supplementary material available at 10.1186/s12890-020-01344-y.

## Background

Chronic obstructive pulmonary disease (COPD) and lung cancer are associated diseases, sharing tobacco as a common cause. Individuals with COPD are two times more likely to develop lung cancer than those without COPD, and individuals with emphysema on CT scan are also at higher risk [1–3]. A common pathophysiology may in part be founded on genetic susceptibility, as exemplified by two single nucleotide polymorphisms in the α-nicotinic acetylcholine receptor (CHRNA 3/5) locus [4], but also more broadly through commonalities in oxidative stress, chronic inflammation, and changes in matrix proteinases [5, 6]. While there are global variations in prevalence, up to one in four North Americans may be diagnosed with COPD in their lifetime [7, 8]. Despite this, there is strong evidence of underdiagnosis of COPD in the primary care population [9, 10] as well as in patients who have lung cancer [11, 12].

Screening of asymptomatic individuals for COPD is not currently recommended by the US Preventative Services Task Force as clinical benefit has not been demonstrated in this population [13]. Conversely, based largely on the results of the National Lung Screening Trial, low-dose computed tomography (LDCT) screening for lung cancer is recommended by the US Preventative Services Task Force and funded by the Centers for Medicare and Medicaid [14–16]. Further momentum to implement screening is provided by the mortality reduction seen in the recently published, large, randomized, NELSON screening trial [17]. A significant proportion of ever smokers is found to have pulmonary emphysema on their screening low-dose CT scan (LDCT), although CT scanning alone is not sufficient to make a diagnosis of COPD [18]. While there is no disease-modifying treatment for smoking-induced COPD, treatment of individuals with moderate or worse COPD with long-acting bronchodilators with or without inhaled corticosteroids has been shown to improve lung function, improve quality of life, and decrease disease exacerbations [19].

COPD is frequently underdiagnosed in the general population [20]. Notably, the incidence of COPD exacerbation-like events has been found to be increased in both diagnosed and undiagnosed groups and health service use for exacerbation events was similarly increased in both groups [20]. Furthermore, in the NHANES III study, although undiagnosed COPD subjects appear healthier than those with a diagnosis, their risk of death was increased compared with subjects without obstruction and that the risk of death may be influenced by lung function [21]. The prevalence of undiagnosed or under-reported COPD in a lung cancer screening population when a risk prediction model such as the PLCOm2012 that incorporates questions on a personal history of COPD is used to assess lung cancer risk is not known [22, 23]. We analyzed the Pan-Canadian Early Detection of Lung Cancer (PanCan) Study data to evaluate the frequency of diagnosed and undiagnosed COPD in a population undergoing lung cancer screening using the PanCan prediction model, a precursor to the PLCOm2012 model, to assess whether spirometry should be routinely performed in lung cancer screening [24].

## Methods

The PanCan study was a single arm lung cancer screening study which recruited from September, 2008, to December, 2010, in 8 Canadian centers. The study was approved at McMaster University by the Hamilton Integrated Research Ethics Board (project 08–367) and by the local ethics board at each study site. Candidates were screened for eligibility using the PanCan model, a prototype of the PLCOm2012 model, which included age (50–75 required), sex, smoking history, family history of lung cancer, personal history of chronic obstructive pulmonary disease, chest X-ray within 3 years, education level, and body-mass index, with the requirement for a 6-year risk of lung cancer ≥2% [24, 25]. Candidates were excluded for significant pre-existing cardiopulmonary conditions, prior lung cancer, other recent cancers, smoking cessation for greater than 15 years, pregnancy, or CT scan within 2 years. Consenting and eligible subjects undertook a detailed health and high-risk occupational exposure questionnaire (listed in Additional file 1: e-Appendix 1), spirometry, and LDCT of the chest. The study included autofluorescence bronchoscopy and blood biomarkers, which are not evaluated here [26]. The study achieved a 6.5% cancer incidence over a median 5.5 years of follow-up [24].

Spirometry was undertaken according to American Thoracic Society recommendations with central quality assurance of spirometry tracings [27]. COPD was defined as “definite” with a forced expiratory volume (first second) (FEV_1_) to forced vital capacity (FVC) ratio of < 0.7 post-bronchodilator. COPD was defined as “probable” with a pre-bronchodilator FEV_1_/FVC < 0.7 if no post-bronchodilator value was available and there was no prior diagnosis of asthma, or “uncertain” with a pre-bronchodilator FEV_1_/FVC < 0.7 if no post-bronchodilator value was available and there was a prior diagnosis of asthma.

LDCT was conducted with minimum section collimation of 1.25 mm, at least 4 data acquisition channels, at 120 kV, 40–50 mA, beam pitch 1.5 to achieve an effective dose of < 2 mSv. Lung parenchyma was reconstructed with a high spatial frequency algorithm and an intermediate spatial frequency algorithm was used for mediastinal structures.

The radiologists’ visual assessment of the extent of emphysema was recorded on a five-point scale (none, minimal, mild, moderate, and severe) and spatial distribution was recorded using a four-point scale (upper, mid, lower, or diffuse) [28].

### Statistics

The primary outcome was a diagnosis (definite, probable, uncertain or no evidence) of COPD based on spirometry. Patients were classified as having of COPD if they had definite or probable COPD. Amongst these patients, severity of COPD was graded using the Global Initiative for Chronic Obstructive Lung Disease (GOLD) criteria.

Summary statistics were used to describe subject characteristics for the population as a whole, and by whether they self-reported a prior diagnosis of COPD. The χ^2^ test and Wilcoxon rank sum test were used to compare the self-reported diagnosis of COPD with patient characteristics, symptoms, prior imaging frequency and radiologist diagnosis of COPD. Logistic regression analyses were used to evaluate prognostic ability of factors on COPD diagnosis as defined by spirometry. Backward selection was used to construct a recommended multivariable model of factors. Residual plots were inspected to assess for non-linear associations and goodness of fit. Discrimination ability was assessed using the concordance statistic. The model was assessed for clinical utility by calculating the risk score as determined by the recommended multivariable model and comparing the risk score with actual risk of COPD. For ease of interpretation, patients were categorized by risk score into deciles. Bootstrapping was then performed to evaluate internal validity of the model based on 2000 bootstrap samples. All estimates and tests were two-sided and statistical significance was defined as a *p*-value ≤0.05.

## Results

Among 2537 subjects recruited to the PanCan study, 2514 had available spirometry data and were included in the analysis. Of these, 527 self-reported a prior diagnosis of COPD and 1987 did not (Table 1). Those reporting a prior diagnosis of COPD were more likely to be female (52 vs 42.7%), were less likely to have completed a secondary school education (14.5 vs 22.2%) or completed post-secondary education (41.6 vs 48.6%), and had a higher mean pack-year smoking history (52.5 vs. 50 years) (all *p* < 0.001). A reported prior COPD diagnosis also conferred a higher likelihood of reporting symptoms of dyspnea, cough, phlegm, or wheeze, a greater likelihood of having one or more chest X-rays in the last 3 years (77 vs 54.9%), and a more common history of other respiratory disease (asthma, pneumonia, respiratory failure) (all *p* < 0.001).

**Table 1:** Population Characteristics by Prior COPD Diagnosis

Characteristic	Overall Population (***N*** = 2514)	Prior Diagnosis COPD (***N*** = 527)	No Prior Diagnosis COPD (***N*** = 1987)	***p***-value*
Age, mean (std dev)	62.3 (5.8)	62.1 (6.1)	62.4 (5.8)	0.38
Gender, male, N (%)	1391 (55.3)	253 (48.0)	1138 (57.3)	< 0.001
Education < secondary	405 (16.1)	117 (22.2)	288 (14.5)	< 0.001
-secondary school	924 (36.8)	191 (36.3)	733 (36.9)	
-post-secondary	1185 (47.1)	219 (41.6)	966 (48.6)	
Pack Years, mean (range)	50 (2.2, 230)	52.5 (2.4, 230)	50 (2.2, 169)	< 0.001
Lung Cancer Risk mean (range)	3.4 (2.0, 38.2)	4.4 (2.0, 34.4)	3.2 (2.0, 38.2)	< 0.001
Current Smoker, n (%)	1566 (62.3)	305 (57.9)	1261 (63.5)	0.020
Dyspnea, n (%)	1133 (45.1)	394 (74.8)	739 (37.2)	< 0.001
Cough, n (%)	1316 (52.4)	361 (68.5)	955 (48.1)	< 0.001
Phlegm, n (%)	1161 (46.2)	329 (62.4)	832 (41.9)	< 0.001
Wheeze, n (%)	943 (37.5)	316 (60.0)	627 (31.6)	< 0.001
Any High Risk Occupation, n (%)	884 (35.2)	198 (37.6)	686 (34.5)	0.20
CXR within 3 years = 0	1017 (40.5)	121 (23.0)	896 (45.1)	< 0.001
= 1	891 (35.4)	212 (40.2)	679 (34.2)	
≥ 2	606 (24.1)	194 (36.8)	412 (20.7)	
CT within 3 years = 0	2441 (97.1)	512 (97.2)	1929 (97.1)	0.86
= 1	68 (2.7)	13 (2.5)	55 (2.8)	
= 2	5 (0.2)	2 (0.4)	3 (0.2)	
Asthma, n (%)	244 (9.7)	117 (22.2)	127 (6.4)	< 0.001
Pulmonary Fibrosis, n (%)	3 (0.1)	1 (0.2)	2 (0.1)	0.51
Pneumonia, n (%)	672 (26.7)	225 (42.7)	447 (22.5)	< 0.001
Respiratory Failure, n (%)	11 (0.4)	8 (1.5)	3 (0.2)	< 0.001

*Comparison of prior diagnosis of COPD to no prior diagnosis of COPD by chi-square (categorical variables) and Wilcoxon rank sum test (continuous)

-Education was collected on a 7 level scale, but grouped here according to secondary and post-secondary completion

In the overall population, spirometry defined COPD was found in 1136 individuals (45.2%), including 833 (41.9%) of those with no prior diagnosis of COPD (Table 2). Among those who did not report a prior diagnosis of COPD, 53.8% of new, spirometry-based COPD diagnoses were classified as moderate or worse severity according to GOLD criteria. Conversely, among those who reported a prior diagnosis of COPD, 32.2% did not meet spirometry criteria for COPD.

**Table 2:** Table of Prior Known COPD status vs. Spirometry COPD Diagnosis

Characteristic	Overall Population (***N*** = 2514)	Prior Diagnosis of COPD (***N*** = 527) n (%)	No prior diagnosis of COPD (***N*** = 1987) n (%)	***p***-value*
**COPD by Spirometry:**				< 0.001
Definite/Probable	1136 (45.2)	303 (57.5)	833 (41.9)	
Uncertain	107 (4.3)	54 (10.3)	53 (2.7)	
None	1271 (50.6)	170 (32.2)	1101 (55.4)	
**Severity of Spirometry Diagnosis COPD (among definite/probable)**				< 0.001
*Stage I (Mild)*	456 (40.1)	71 (23.4)	385 (46.2)	
*Stage II (Moderate)*	583 (51.3)	173 (57.1)	410 (49.2)	
*Stage III (Severe)*	87 (7.7)	51 (16.8)	36 (4.3)	
*Stage IV (Very Severe)*	10 (0.9)	8 (2.6)	2 (0.2)	

*Comparison of prior diagnosis of COPD to no prior diagnosis of COPD by chi-square

**Fig. 1:**
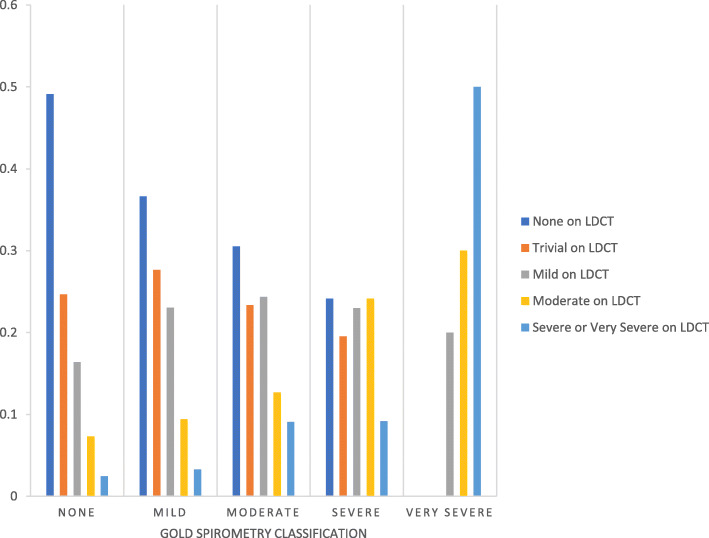
COPD severity as determined by low-dose CT according to GOLD classification by spirometry. Footnote to figure: -Due to small numbers, low-dose CT (LDCT) groupings of severe (*n* = 97) and very severe (*n* = 18) COPD were combined. -Weighted Kappa = 0.16

The relationship between COPD diagnosed by spirometry and emphysema severity reported by LDCT was poor (Weighted Kappa =0.16) (Fig. 1). Among 1378 individuals having no COPD by spirometry, 361 (26.2%) had mild or worse emphysema by LDCT report. By contrast, among 97 individuals with severe or very severe disease by spirometry, 38 (39.2%) had no or trivial COPD by LDCT report.

Table 3 shows the factors associated with a prior self-reported COPD diagnosis (irrespective of spirometry diagnosis). In the multivariable model, symptoms of dyspnea, wheeze, cough and phlegm, number of comorbidities, and being an ex-smoker were all associated with having a prior diagnosis of COPD. Female sex, a lower average education level, greater pack-year smoking history, and chest x-ray testing were associated with COPD only on univariable analysis.

**Table 3:** Factors Associated with a Prior Diagnosis of COPD

Characteristic	Odds Ratio (95% CI)	***p***-value
**UNIVARIABLE MODEL**		
Age */ year*	0.99 (0.98, 1.01)	0.29
Sex *Male* vs *Female*	0.69 (0.57, 0.84)	< 0.001
Education Level */ unit*	0.89 (0.84, 0.94)	< 0.001
Age Started Smoking */ year*	0.98 (0.95, 1.01)	0.24
Average Cigarettes / Day Smoked (*Log-transformed)*	1.94 (1.53, 2.47)	< 0.001
Pack Years *(Log-transformed)*	1.81 (1.43, 2.29)	< 0.001
Presently a Smoker *Yes* vs *No*	0.79 (0.65, 0.96)	0.019
Serious Attempt to Quit (of those who are presently a smoker) *Yes* vs *No*	1.09 (0.74, 1.62)	0.66
# of Healthcare Professionals Asking About Smoking (of those who are presently a smoker) */ time*	1.90 (1.41, 2.56)	< 0.001
Dyspnea *Yes* vs *No*	5.00 (4.03, 6.21)	< 0.001
Cough *Yes* vs *No*	2.35 (1.92, 2.88)	< 0.001
Phlegm *Yes* vs *No*	2.31 (1.89, 2.81)	< 0.001
Wheeze *Yes* vs *No*	3.25 (2.66, 3.96)	< 0.001
Any High Risk Occupation *Yes* vs *No*	1.14 (0.94, 1.39)	0.19
Chest X-Rays *≥1* vs *0*	2.76 (2.21, 3.44)	< 0.001
CT Scans *≥1* vs *0*	0.98 (0.55, 1.73)	0.93
Number of Comorbidities *≥1* vs *0*	2.57 (2.04, 3.25)	< 0.001
**MULTIVARIABLE MODEL**		
Dyspnea *Yes* vs *No*	3.38 (2.67, 4.27)	< 0.001
Wheeze *Yes* vs *No*	1.86 (1.48, 2.35)	< 0.001
Cough *Yes* vs *No*	1.47 (1.13, 1.91)	0.004
Phlegm *Yes* vs *No*	1.35 (1.05, 1.74)	0.019
Number of Comorbidities *≥1* vs *0*	1.98 (1.54, 2.56)	< 0.001
Presently a Smoker *Yes* vs *No*	0.53 (0.42, 0.66)	< 0.001

-Comorbidities include coronary artery disease, angina, myocardial infarction, congestive heart failure, peripheral vascular disease, asthma, pneumonia, respiratory failure, and any cancer

-Education was collected on a 7 level/unit scale, but grouped in Table 1 according to secondary and post-secondary completion

In assessing factors associated with COPD by spirometry criteria, following backward selection, the final multivariable model included age, current smoking status, number of pack-years, presence of dyspnea, wheeze, participation in a high-risk occupation, and emphysema extent on LDCT (Table 4). The c-statistic, which is a measure of discriminatory ability, was 0.627 (95% CI = 0.607 to 0.650), which is generally considered poor discrimination.

**Table 4:** Predictive factors of COPD defined by spirometry

Characteristic	Odds Ratio (95% CI)	***p***-value
**UNIVARIABLE MODEL**		
Age */ year*	1.05 (1.03, 1.06)	< 0.001
Gender *Male* vs *Female*	1.39 (1.19, 1.63)	< 0.001
Education Level */ unit*^a^	0.97 (0.92, 1.01)	0.14
Age Started Smoking */ year*	1.01 (0.98, 1.03)	0.71
Average Cigarettes / Day Smoked (*Log-transformed)*	1.24 (1.03, 1.49)	0.024
Pack Years *(Log-transformed)*	1.49 (1.24, 1.79)	< 0.001
Presently a Smoker *Yes* vs *No*	1.25 (1.06, 1.47)	0.008
Serious Attempt to Quit (of those who are presently a smoker) *Yes* vs *No*	1.19 (0.88, 1.61)	0.26
# of Healthcare Professionals Asking About Smoking (of those who are presently a smoker) */ time*	0.85 (0.69, 1.06)	0.14
Emphysema Extent by LDCT		
*None*	Reference	< 0.001
*Trivial*	1.52 (1.24, 1.86)	
*Mild*	2.20 (1.77, 2.74)	
*Moderate*	2.58 (1.94, 3.44)	
*Severe*	3.76 (2.42, 5.85)	
*Very Severe*	14.76 (3.38, 64.47)	
Emphysema Distribution by LDCT *None*		
*Diffuse*	Reference	< 0.001
*Lower Lobe*	1.60 (1.24, 2.06)	
*Upper Lobe*	1.68 (0.73, 3.83)	
	2.19 (1.84, 2.60)	
Dyspnea *Yes* vs *No*	1.55 (1.32, 1.81)	< 0.001
Cough *Yes* vs *No*	1.23 (1.05, 1.44)	0.010
Phlegm *Yes* vs *No*	1.19 (1.02, 1.40)	0.028
Wheeze *Yes* vs *No*	1.43 (1.21, 1.68)	< 0.001
Any High Risk Occupation *Yes* vs *No*	1.25 (1.06, 1.47)	0.008
Chest X-Rays *≥1* vs *0*	1.24 (1.05, 1.45)	0.010
CT Scans *≥1* vs *0*	1.99 (1.23, 3.21)	0.005
Number of Comorbidities^b^ *≥1* vs *0*	1.03 (0.83, 1.28)	0.77
**MULTIVARIABLE MODEL**		
Emphysema Extent		
*None*	Reference	< 0.001
*Trivial*	1.51 (1.23, 1.86)	
*Mild*	2.05 (1.64, 2.57)	
*Moderate*	2.37 (1.77, 3.18)	
*Severe*	3.09 (1.97, 4.86)	
*Very Severe*	10.66 (2.40, 47.37)	
Age */ year*	1.06 (1.04, 1.07)	< 0.001
Dyspnea *Yes* vs *No*	1.33 (1.11, 1.59)	0.002
Presently a Smoker *Yes* vs *No*	1.38 (1.15, 1.65)	< 0.001
Pack Years *(Log-transformed)*	1.34 (1.10, 1.63)	0.003
Wheeze *Yes* vs *No*	1.25 (1.04, 1.51)	0.020
Any High Risk Occupation *Yes* vs *No*	1.21 (1.02, 1.44)	0.030

^a^Education was collected on a 7 level/unit scale, but grouped in Table 1 according to secondary and post-secondary completion

^b^Comorbidities include coronary artery disease, angina, myocardial infarction, congestive heart failure, peripheral vascular disease, asthma, pneumonia, respiratory failure, and any cancer

Despite their association with a prior diagnosis of COPD and prediction of a spirometry-based diagnosis of COPD, only 51.1% (579/1133) of patients with dyspnea and 50.7% (478/943) with wheeze met the GOLD criteria for diagnosis, while 37.7% (245/650) of subjects having no respiratory symptom also met the criteria for COPD. Similarly, 47.3% (740/1566) of current smokers met GOLD criteria for COPD, as did 41.8% (396/948) of former smokers.

Table 5 shows the actual risk of COPD based on model predicted risk deciles. Those in the lowest predicted risk decile still had an actual observed rate of COPD of 27.4%. There is a gradual increase in the rate of COPD by decile to a rate of 75.0% in the highest risk decile. Calibration is assessed by how closely the predicted estimate is with the observed estimate. In two of the ten deciles, the predicted estimate falls outside the range of the 95% bias-corrected and accelerated (BCa) confidence intervals, which is calculated via bootstrapping.

**Table 5:** Actual COPD risk by model risk decile

Decile	Predicted Estimate	N	N (%) with COPD	95% BCa Bootstrap CI
1	27.8%	252	69 (27.4)	21.4–35.5
2	33.7%	251	77 (30.7)	24.7–36.7
3	37.4%	251	83 (33.1)	26.7–38.7
4	40.4%	251	80 (31.9)	25.9–37.2
5	43.4%	252	108 (42.9)	36.1–48.8
6	46.3%	251	117 (46.6)	40.2–52.5
7	49.5%	251	127 (50.6)	43.8–56.2
8	53.0%	252	143 (56.8)	51.2–62.7
9	57.5%	251	143 (57.0)	50.6–62.2
10	65.3%	252	189 (75.0)	69.4–80.2

## Discussion

With data now supporting LDCT screening for lung cancer, a large population of tobacco users may now have contact with screening programs [14]. This offers the opportunity to consider a wider use of such programs to improve the health of this population. The most obvious add-on to such programs has been tobacco cessation. The lung cancer screening population, including those ineligible for trials, express interest in smoking cessation [29, 30]. Smoking cessation interventions are highly cost effective [31] and provide survival benefits likely to exceed the benefit of screening itself [32].

The present work suggests that the lung cancer screening population would also benefit from concurrent screening for COPD by spirometry. The association between COPD and lung cancer has already been demonstrated [1, 2]. COPD is a factor in modeling risk for lung cancer [23], and COPD has also been shown to have a higher prevalence in a lung cancer population [33]. At the end of life, individuals with COPD have care needs comparable to individuals with lung cancer [34, 35], and COPD confers a significant economic burden [36, 37]. Appropriate management of individuals with COPD is likely to improve quality of life at a reasonable cost [38–41].

In the PanCan study, the prevalence of COPD was 45.2% as defined by spirometry. This is slightly higher than that observed in the ACRIN population of the National Lung Screening Trial (34.4%) and the NELSON screening study (38.3%) [18, 42]. While the difference is not readily explained by the relative age and smoking history of the cohorts, a prior history of COPD was used in the risk model of the PanCan study. In the PanCan population without a prior diagnosis of COPD, 41.9% met criteria by spirometry. Among those diagnosed with COPD, 59.9% had moderate or worse disease.

Conversely, 32.2% of subjects reporting a diagnosis of COPD did not meet spirometry criteria for a COPD diagnosis. Although we do not know what portion of these individuals previously had spirometry, Fernandez-Villar et al. found that 21.6% of those undergoing spirometry were incorrectly diagnosed as having COPD despite showing a non-obstructive pattern [43]. In our study, individuals with self-reported COPD more commonly reported respiratory symptoms, symptoms which might have served as diagnostic triggers for clinicians. The fact that such individuals were also more likely to be ex-smokers raises the question of whether their symptoms, reported COPD diagnosis, or resulting medical care motivated tobacco cessation. While by GOLD definition these patients were misdiagnosed, emerging data suggests that half of current and former smokers not meeting spirometry criteria may suffer respiratory symptoms, with an increase in respiratory exacerbations and a loss quality of life [44, 45]. It is presently unclear how to address the needs of this population.

CT changes of emphysema were reported in just over half of individuals (50.9%) not having COPD according to spirometry. Previous investigators have found that a portion of individuals diagnosed with COPD by CT do not meet criteria by spirometry [46, 47]. In a large population with respiratory symptoms not meeting spirometry criteria, Regan et al. found that 42.3% had CT evidence of either emphysema (24.0%) or airway thickening (30.7%) [44]. The reason for this apparent mismatch between radiologic and spirometric findings is not clear. Given that other studies have made similar findings, there is likely a population for whom the changes observed on CT are physiologically insufficient to make a formal diagnosis of COPD possible. This is consistent with data which suggests that early radiologic changes presage later changes in spirometry [48]. Certainly, the extent to which CT changes are detected in individuals without COPD by spirometry will depend on which CT changes are sought, as exemplified by the work of Regan et al. [44].

In our population, the relationship between severity of COPD by LDCT as compared to FEV_1_ was poor, although LDCT did contribute to COPD prediction in our model. By comparison, in another low-dose CT screening population, Omori et al. found a modest association between a visual, semi-quantitative emphysema score and spirometry findings [47]. While data from the COPDGene study showed that subjective readings of emphysema in standard dose CT imaging correlated well with quantitative results and spirometry [49], other data suggests radiologists are more likely to overestimate COPD than would a CT densitometry algorithm [28].

To improve sensitivity for emphysema, investigators in Japan added a single-slice high resolution CT of the upper lung field to a low-dose CT scan screening program for lung cancer. Of note, 100 (16%) of 615 subjects were never smokers. Using visual classification, investigators increased the detection of low attenuation from 6.4% with LDCT alone to 23.3% with a HRCT slice [50].

Mets et al. published a large (*n* = 1140) single centre analysis of patients from the NELSON trial [18]. CT diagnosis of emphysema was based on percentage of lung attenuation below − 950 Hounsfield units and air trapping was assessed with expiratory CT views. A model incorporating the CT factors plus body mass index, pack-years of smoking, and current smoking status had a ROC curve AUC of 0.83 (95% CI, 0.81–0.86), with sensitivity of 63% and a specificity of 88% to detect COPD as compared with pre-bronchodilator spirometry.

Our study was conducted using LDCT without additional CT maneuvers in a population at higher risk for lung cancer (smoking history median 50 vs. 38 pack-years), a scenario more likely to be adopted by jurisdictions with resource constraints. In this context, our COPD prediction model had poor discrimination.

Importantly, when we assess the PanCan population by COPD risk estimate decile, even the lowest decile still had an estimated risk for COPD of 27.8%, with the top decile having a risk of 65.3%. In light of the limited sensitivity of our and others’ risk models, the COPD risk in our population is arguably sufficient to warrant spirometry testing of all screened patients, regardless of number of risk factors. Our data show that the use of symptoms or current smoking status without spirometry will frequently lead to an incorrect diagnosis of COPD.

This exploratory analysis of a prospective trial has limitations. Prior diagnosis of COPD was based on patient recall, and reported symptoms and history were only captured at baseline; any associations between the two are therefore hypothesis generating. Post-bronchodilator spirometry values were not used in all cases, requiring us to define a group with ‘probable’ COPD. LDCT reading was conducted by experienced and study-trained radiologists, but software analysis was not employed, and interpretation is necessarily subjective. The use of imaging software may enhance COPD diagnosis and could be more cost-effective than additional CT maneuvers. The PanCan cohort was comparatively high risk for lung cancer, and our findings of COPD prevalence may not extrapolate to lower risk screening populations.

## Conclusions

The primary goal of LDCT screening has been to diminish the risk of death from lung cancer. It has been recognized that screening also provides an opportunity for a smoking cessation intervention. The present study demonstrates that being eligible for high-risk lung cancer screening confers a substantial risk of having underlying COPD. While the presence of clinical factors and emphysema on LDCT are somewhat predictive of COPD, no subpopulation in our study could be considered as low risk. For those conducting LDCT screening for lung cancer in a high-risk population, consideration should be given to universal spirometric assessment for COPD.

## Supplementary Information

**Additional file 1.**

**Additional file 2.**

## Data Availability

The dataset analysed during the current study are available from the corresponding author on reasonable request.

## Abbreviations

COPD: Chronic obstructive pulmonary disease
FEV1: Forced expiratory volume (first second)
FVC: Forced vital capacity
GOLD: Global Initiative for Chronic Obstructive Lung Disease
LDCT: Low-dose CT scan
