# Supplementary material for: Chronic obstructive pulmonary disease prevalence and prediction in a high-risk lung cancer screening population
Source: BMC Pulm Med. 2020 Nov 16;20:300. doi: 10.1186/s12890-020-01344-y (PMC7670711; doi:10.1186/s12890-020-01344-y)
Supplement: Supplementary file 1 — Additional file 1. [file 12890_2020_1344_MOESM1_ESM.docx]

**e-Appendix 1**

List of High-Risk occupations on the baseline study questionnaire:

[Q] Have you ever worked in any of the following occupations or had the following occupational exposures?

[Q101a] Mining, either on the surface or underground?

[Q102a] With asbestos, in any industry?

[Q103a] With arsenic?

[Q104a] With chromium?

[Q105a] With uranium?

[Q106a] With radium?

[Q107a] In a smelter?

[Q108a] In a steel mill?

[Q109a] In a coke oven?

[Q110a] In a foundry?

[Q111a] In a nuclear power plant?

[Q112a] In roofing?

[Q113a] With tars or asphalt?

[Q114a] In construction?

[Q115a] In road construction?

[Q116a] Exposed to diesel fuel or exhaust fumes?
