# Supplementary material for: Chronic obstructive pulmonary disease prevalence and prediction in a high-risk lung cancer screening population
Source: BMC Pulm Med. 2020 Nov 16;20:300. doi: 10.1186/s12890-020-01344-y (PMC7670711; doi:10.1186/s12890-020-01344-y)
Supplement: Supplementary file 2 — Additional file 2. [file 12890_2020_1344_MOESM2_ESM.docx]

**e-Appendix 2**

Research Ethics Boards at each study site

Hamilton Integrated Research Ethics Board, Hamilton, Ontario, for the Juravinski Cancer Centre site

Calgary Health Research Ethics Board, Calgary, Alberta, for the Foothills Hospital site

Nova Scotia Health Authority Research Ethics Board, Halifax, Nova Scotia, for the Queen Elizabeth II Health Sciences Centre site

Ottawa Health Science Network Research Ethics Board, Ottawa, Ontario, for the Ottawa Hospital site

Comité d’éthique de la recherche IUCPQ-UL, Québec, Québec, for l’Institut universitaire de cardiologie et pneumonolgie de Québec site

Newfoundland and Labrador Health Research Ethics Authority, St. John’s, Newfoundland, for the Health Sciences Centre General Hospital site

University Health Network Research Ethics Board C, Toronto, Ontario, for the Princess Margaret Cancer Centre site

BCCA Research Ethics Board, Vancouver, British Columbia, for the British Columbia Cancer Research Centre site
